# Properties of the symmetric difference in lattices with complementation

**DOI:** 10.1515/ms-2026-0136

**Published:** 2026-04-21

**Authors:** Václav Cenker, Ivan Chajda, Helmut Länger

**Affiliations:** Department of Algebra and Geometry, Faculty of Science, Palacký University Olomouc, 17. Listopadu 12, CZ-771 46 Olomouc, Czech Republic; Institute of Discrete Mathematics and Geometry, Faculty of Mathematics and Geoinformation, 2027259 TU Wien, Wiedner Hauptstrasse 8–10, A-1040 Vienna, Austria

**Keywords:** lattice with complementation, symmetric difference, De Morgan’s laws, distributivity, associativity, free algebra, Primary 06D05, Secondary 06B05, 06B20, 06F25, 06C15, 06E20

## Abstract

The symmetric difference in Boolean lattices can be defined in two different but equivalent forms. However, it can be introduced also in every bounded lattice with complementation where these two forms need not coincide. We study lattices with complementation and the variety of such lattices where these two expressions coincide and point out explicitly some interesting subvarieties. Using a result of J. Berman we estimate the size of free algebras in these subvarieties. It is well-known that the symmetric difference is associative in every Boolean lattice. We prove that it is just the property of Boolean lattices, namely the symmetric difference in a lattice with complementation is associative if and only if this lattice is Boolean. Similarly, we prove that a lattice with complementation is Boolean if and only if the symmetric difference satisfies a certain simple identity in two variables. We also characterize lattices with a unary operation satisfying De Morgan’s laws.

## 1 Introduction

The aim of this paper is to show several important properties of the symmetric difference in complemented lattices where the complementation is a unary operation. Because the symmetric difference can be defined in two different ways which coincide in Boolean lattices, we are interested in lattices which need not be distributive but these two forms of symmetric difference coincide. It is well-known that in a Boolean algebra the symmetric difference is associative. We show that also, conversely, associativity of the symmetric difference yields distributivity of the complemented lattice in question.

Let us mention that for orthomodular and for orthocomplemented lattices the symmetric difference was intensively investigated by several authors, see e.g. [1], [2], [3].

We start with definitions of some basic concepts.

Let **L** = (*L*, ∨, ∧, 0, 1) be a bounded lattice and *a* ∈ *L*. An element *b* of *L* is called a *complement* of *a* if *a* ∨ *b* = 1 and *a* ∧ *b* = 0. The *lattice* **L** is called *complemented* if every element of *L* has a complement which need not be unique. Let ′ be a unary operation on *L*. Then ′ is called

(i) *antitone* if, for all *x*, *y* ∈ *L*, *x* ≤ *y* implies *y*′ ≤ *x*′,
(ii) an *involution* if *x*″ = *x* for all *x* ∈ *L*,
(iii) a *complementation* if, for every *x* ∈ *L*, *x* ∨ *x*′ = 1 and *x* ∧ *x*′ = 0.

In (iii), *x*′ is a complement of *x* for every *x* ∈ *L*. The algebra **L** = (*L*, ∨, ∧, ′, 0, 1) where ′ is a complementation will be called a *lattice with complementation*. If, moreover, ′ is an antitone involution then **L** is called an *ortholattice*.

At first we prove an auxiliary result using identities similar to those studied in refs. [4], [5].

**Lemma 1.1.**

Let **L** = (*L*, ∨, ∧, ′, 0, 1) be a lattice with complementation satisfying one of the following identities:

(i) *x* ∧ *y* ≈ *x* ∧ (*x*′ ∨ *y*),
(ii) *x* ∨ *y* ≈ *x* ∨ (*x*′ ∧ *y*).

Then **L** satisfies the identity *x*″ ≈ *x*.

**Proof.**

Assume (i). Then

(1.1) $$x\wedge x^{\prime \prime }\approx x\wedge \left(x^{\prime }\vee x^{\prime \prime }\right)\approx x\wedge 1\approx x.$$

Hence *x*′ ≈ *x*′ ∧ x‴ and

$$x^{\prime \prime \prime }\vee x^{\prime }\approx x^{\prime \prime \prime }\vee \left(x^{\prime }\wedge x^{\prime \prime \prime }\right)\approx x^{\prime \prime \prime }$$

whence

$$x\vee x^{\prime \prime \prime }\approx x\vee x^{\prime \prime \prime }\vee x^{\prime }\approx 1.$$

Using this, (1.1) and (i) we obtain

$$x\approx x\wedge x^{\prime \prime }\approx x^{\prime \prime }\wedge x\approx x^{\prime \prime }\wedge \left(x^{\prime \prime \prime }\vee x\right)\approx x^{\prime \prime }\wedge 1\approx x^{\prime \prime }.$$

By dualizing the preceding proof we see that (ii) implies *x*″ ≈ *x*. □

It is well-known (see e.g. [6]) that in a distributive bounded lattice every element has at most one complement. A distributive lattice with complementation is an ortholattice which is called a *Boolean lattice*. Hence in Boolean lattices **L**, for every *x* ∈ *L*, *x*′ is the unique complement of *x*.

It is evident that if (*L*, ∨, ∧, ′, 0, 1) is a lattice with complementation and *a* ∈ *L* then *a* = 0 if and only if *a*′ = 1 and, conversely, *a* = 1 if and only if *a*′ = 0.

In every bounded lattice **L** = (*L*, ∨, ∧, ′, 0, 1) with a unary operation ′ one can introduce two so-called *symmetric differences*, i.e. the term operations

$$\begin{array}{ll} x{+}_{1}y & ≔\left(x^{\prime }\wedge y\right)\vee \left(x\wedge y^{\prime }\right),\\ x{+}_{2}y & ≔\left(x\vee y\right)\wedge \left(x^{\prime }\vee y^{\prime }\right). \end{array}$$

which coincide if **L** is a Boolean lattice, see e.g. [7]. The question is whether this holds only in Boolean lattices. Hence, in what follows, we are interested in lattices with complementation satisfying the identity

(1.2) $$\left(x^{\prime }\wedge y\right)\vee \left(x\wedge y^{\prime }\right)\approx \left(x\vee y\right)\wedge \left(x^{\prime }\vee y^{\prime }\right).$$

We call this identity the *coincidence identity*. Let us note that varieties of lattices with complementation where the complementation satisfies certain adjointness properties were already studied by the authors in ref. [8].

**Example 1.**

**Figure 1:**
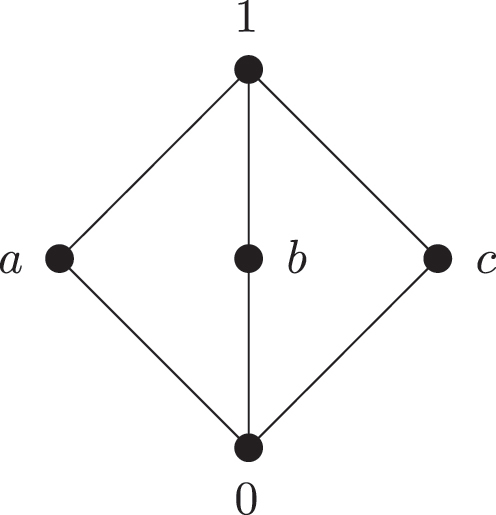
Modular lattice **M**_3_.

Consider the modular lattice **M**_3_ = (*M*_3_, ∨, ∧) depicted in Figure 1.

There are exactly eight possibilities how to define a complementation ′ on **M**_3_. Each of these complementations is antitone, but none is an involution. One such complementation is given by

$$\begin{array}{cccccc} x & 0 & a & b & c & 1\\ x^{\prime } & 1 & b & c & a & 0 \end{array}$$

We have

$$\begin{array}{ll} a{+}_{1}b & =\left(a^{\prime }\wedge b\right)\vee \left(a\wedge b^{\prime }\right)=\left(b\wedge b\right)\vee \left(a\wedge c\right)=b\vee 0=b\neq 1=1\wedge \left(b\vee c\right)\\ & =\left(a\vee b\right)\wedge \left(a^{\prime }\vee b^{\prime }\right)=a{+}_{2}b, \end{array}$$

i.e., the lattice $\left(M_{3},\vee ,\wedge ,{\;}^{\prime },0,1\right)$ with complementation does not satisfy identity (1.2) (cf. Corollary 2.1.1).

For lattices with complementation satisfying identity (1.2) we can show the following result.

**Lemma 1.2.**

Let **L** = (*L*, ∨, ∧, ′, 0, 1) be a lattice with complementation satisfying identity (1.2) and let *a* ∈ *L*. Then the following hold:

(i) **L** satisfies the identity *x*′ ∨ (*x* ∧ *x*″) ≈ 1,
(ii) *a* ∧ *a*″ ≤ *a*′ if and only if *a* = 0.

**Proof.**

(i) Identity (1.2) implies *x*′ ∨ (*x* ∧ *x*″) ≈ (*x*′ ∧ *x*′) ∨ (*x* ∧ *x*″) ≈ (*x* ∨ *x*′) ∧ (*x*′ ∨ *x*″) ≈ 1 ∧ 1 ≈ 1.
(ii) Because of (i) the following are equivalent *a* ∧ *a*″ ≤ *a*′, *a*′ ∨ (*a* ∧ *a*″) = *a*′, *a*′ = 1, *a* = 0. □

Recall that the identities (*x* ∨ *y*)′ ≈ *x*′ ∧ *y*′ and (*x* ∧ *y*)′ ≈ *x*′ ∨ *y*′ for lattices (*L*, ∨, ∧, ′) with a unary operation ′ are called *De Morgan’s laws*.

## 2 Lattices and varieties of lattices satisfying the coincidence identity for symmetric differences

The following lemma presents two identities holding in any ortholattice satisfying identity (1.2).

**Lemma 2.1.**

Let (*L*, ∨, ∧, ′, 0, 1) be an ortholattice satisfying identity (1.2). Then it satisfies the identities

$$\begin{array}{ll} \left(x\wedge y\right)\vee \left(x\wedge y^{\prime }\right)\vee \left(x^{\prime }\wedge y\right)\vee \left(x^{\prime }\wedge y^{\prime }\right) & \approx 1,\\ \left(x\vee y\right)\wedge \left(x\vee y^{\prime }\right)\wedge \left(x^{\prime }\vee y\right)\wedge \left(x^{\prime }\vee y^{\prime }\right) & \approx 0. \end{array}$$

**Proof.**

We have

$$\left(x\wedge y\right)\vee \left(x\wedge y^{\prime }\right)\vee \left(x^{\prime }\wedge y\right)\vee \left(x^{\prime }\wedge y^{\prime }\right)\approx \left(x{+}_{1}y\right)\vee {\left(x{+}_{2}y\right)}^{\prime }\approx \left(x{+}_{1}y\right)\vee {\left(x{+}_{1}y\right)}^{\prime }\approx 1.$$

The second identity is dual to the first one. □

In the previous example, each of the elements *a*, *b*, *c* has two incomparable complements which, moreover, are complements of each other. We can show that there exist lattices satisfying identity (1.2) having an element with two incomparable complements that are not complements of each other, see the following example.

**Example 2.**

**Figure 2:**
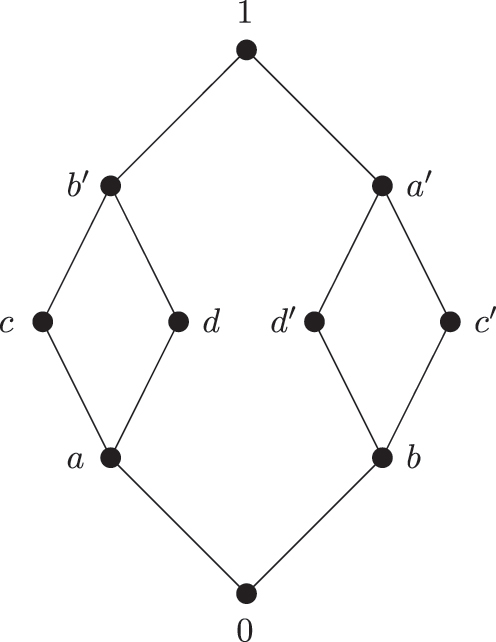
Ortholattice satisfying identity (1.2).

**Figure 3:**
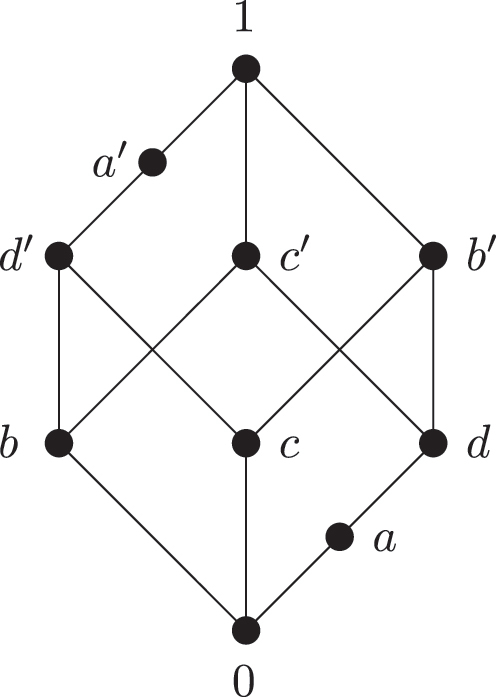
Ortholattice satisfying identity (1.2).

In Figures 2 and 3 ortholattices satisfying identity (1.2) are visualized:

As one can see, in the ortholattice depicted in Figure 2 the elements *c*′ and *d*′ are incomparable complements of *c*, but *c*′ and *d*′ are not complements of each other whereas in the ortholattice visualized in Figure 3 the elements *a*′ and *d*′ are comparable complements of *a*. Moreover, the first ortholattice is not subdirectly irreducible while the second is. The congruence monolith of the second ortholattice contains {*a*, *d*} and {*d*′, *a*′} as non-trivial classes.

However, the situation shown in Example 1 can be generalized as follows.

**Theorem 2.1.**

Let (*L*, ∨, ∧, ′, 0, 1) be a non-trivial lattice with complementation satisfying identity (1.2) and let *a* ∈ *L*. Then there does not exist some *b* ∈ *L* being a complement of *a* and *a*′.

**Proof.**

If there existed some *b* ∈ *L* that was a complement of *a* and *a*′ then using (1.2) we would obtain

$$\begin{array}{ll} a & =a\vee 0\vee \left(a\wedge b^{\prime }\right)=a\vee \left(a^{\prime }\wedge b\right)\vee \left(a\wedge b^{\prime }\right)=a\vee \left(\left(a\vee b\right)\wedge \left(a^{\prime }\vee b^{\prime }\right)\right)\\ & =a\vee \left(1\wedge \left(a^{\prime }\vee b^{\prime }\right)\right)=a\vee a^{\prime }\vee b^{\prime }=1 \end{array}$$

and hence

$$0=b\wedge a=b\wedge 1=b=b\vee 0=b\vee \left(a^{\prime }\wedge a\right)=b\vee \left(a^{\prime }\wedge 1\right)=b\vee a^{\prime }=1,$$

a contradiction. □

**Corollary 2.1.1.**

A lattice (*L*, ∨, ∧, ′, 0, 1) with complementation satisfying identity (1.2) cannot contain a subalgebra isomorphic to $\left(M_{3},\vee ,\wedge ,{\;}^{\prime },0,1\right)$ .

On the other hand, we can show that if *x* and *y* or *x* and *y*′ are comparable for all *x*, *y* ∈ *L* then the ortholattice **L** satisfies identity (1.2).

**Theorem 2.2.**

Let **L** = (*L*, ∨, ∧, ′, 0, 1) be an ortholattice having the property that for all *x*, *y* ∈ *L*, either *x* and *y* or *x* and *y*′ are comparable with each other. Then **L** satisfies identity (1.2).

**Proof.**

Let *a*, *b* ∈ *L*.

If *a* ≤ *b* then *b*′ ≤ *a*′ and hence *a* ∧ *b*′ ≤ *b* ∧ *a*′ and

$$a{+}_{1}b=\left(a^{\prime }\wedge b\right)\vee \left(a\wedge b^{\prime }\right)=\left(b\wedge a^{\prime }\right)\vee \left(a\wedge b^{\prime }\right)=b\wedge a^{\prime }=\left(a\vee b\right)\wedge \left(a^{\prime }\vee b^{\prime }\right)=a{+}_{2}b.$$

In the three remaining cases the equality *a* +_1_ *b* = *a* +_2_ *b* can be proved analogously by using the symmetric role of *a* and *b*, respectively of *a*, *b* and their complements. □

Recall that the *horizontal sum* of a family (*C*_*i*_, ≤_*i*_, 0, 1), *i* ∈ *I*, of bounded chains with *C*_*i*_ ∩ *C*_*j*_ = {0, 1} for all *i*, *j* ∈ *I* with *i* ≠ *j* is the bounded lattice

$$\left(\underset{i\in I}{⋃}C_{i},\underset{i\in I}{⋃}\leq_{i},0,1\right).$$

The complementation in horizontal sums of chains is described in the next theorem.

**Theorem 2.3.**

Let **C**_1_ = (*C*_1_, ≤, 0, 1) and **C**_2_ = (*C*_2_, ≤, 0, 1) be bounded chains satisfying *C*_1_ ∩ *C*_2_ = {0, 1}, let **L** = (*L*, ∨, ∧, 0, 1) denote the horizontal sum of **C**_1_ and **C**_2_ and let ′ be a unary operation on *L*. Then the following holds:

(i) The operation ′ is a complementation if and only if 0′ = 1, 1′ = 0, (*C*_1_ \ {0, 1})′ ⊆ *C*_2_ \ {0, 1} and (*C*_2_ \ {0, 1})′ ⊆ *C*_1_ \ {0, 1},
(ii) if ′ is a complementation then **L** satisfies identity (1.2),
(iii) if ′ is a complementation then **L** satisfies De Morgan’s laws if and only if ′ is antitone.

**Proof.**

Let *a*, *b* ∈ *L*.

(i) First assume ′ to be a complementation. As mentioned in the beginning, we have 0′ = 1 and 1′ = 0. Moreover, if *a* ∈ *C*_1_ \ {0, 1} then *a*′ ∈ *L* \ *C*_1_ = *C*_2_ \ {0, 1} showing (*C*_1_ \ {0, 1})′ ⊆ *C*_2_ \ {0, 1}. By symmetry, (*C*_2_ \ {0, 1})′ ⊆ *C*_1_ \ {0, 1}. Conversely, if 0′ = 1, 1′ = 0, (*C*_1_ \ {0, 1})′ ⊆ *C*_2_ \ {0, 1} and (*C*_2_ \ {0, 1})′ ⊆ *C*_1_ \ {0, 1} then ′ is a complementation, namely the equalities *a* ∨ *a*′ = 1 and *a* ∧ *a*′ = 0 can be seen by distinguishing the cases *a* = 0, *a* = 1, *a* ∈ *C*_1_ \ {0, 1} and *a* ∈ *C*_2_ \ {0, 1}.
(ii) If *a* ∈ {0, 1} or *b* ∈ {0, 1} then *a* +_1_ *b* = *a* +_2_ *b*. If *a*, *b* ∈ *C*_1_ \ {0, 1} then *a* ∨ *b* ∈ *C*_1_ \ {0, 1} and *a*′ ∨ *b*′ ∈ *C*_2_ \ {0, 1} and hence
$$a{+}_{1}b=\left(a^{\prime }\wedge b\right)\vee \left(a\wedge b^{\prime }\right)=0\vee 0=0=\left(a\vee b\right)\wedge \left(a^{\prime }\vee b^{\prime }\right)=a{+}_{2}b.$$
  If *a* ∈ *C*_1_ \ {0, 1} and *b* ∈ *C*_2_ \ {0, 1} then *a*′ ∧ *b* ∈ *C*_2_ \ {0, 1} and *a* ∧ *b*′ ∈ *C*_1_ \ {0, 1} and hence
$$a{+}_{1}b=\left(a^{\prime }\wedge b\right)\vee \left(a\wedge b^{\prime }\right)=1=1\wedge 1=\left(a\vee b\right)\wedge \left(a^{\prime }\vee b^{\prime }\right).$$
  The remaining cases can be treated similarly.
(iii) Assume ′ to be a complementation. If **L** satisfies De Morgan’s laws and *a* ≤ *b* then *b*′ = (*a* ∨ *b*)′ = *a*′ ∧ *b*′ ≤ *a*′. This shows that ′ is antitone. Conversely, assume ′ to be antitone. If *a*, *b* ∈ *C*_1_ and *a* ≤ *b* then (*a* ∨ *b*)′ = *b*′ = *b*′ ∧ *a*′. If *a* ∈ *C*_1_ \ {0, 1} and *b* ∈ *C*_2_ \ {0, 1} then (*a* ∨ *b*) = 1′ = 0 = *a*′ ∧ *b*′. The other cases can be treated similarly. Hence **L** satisfies the identity (*x* ∨ *y*)′ ≈ *x*′ ∧ *y*′. Dual arguments show that **L** satisfies also the identity (*x* ∧ *y*)′ ≈ *x*′ ∨ *y*′. □

We apply Theorem 2.3 in the following cases.

**Example 3.**

**Figure 4:**
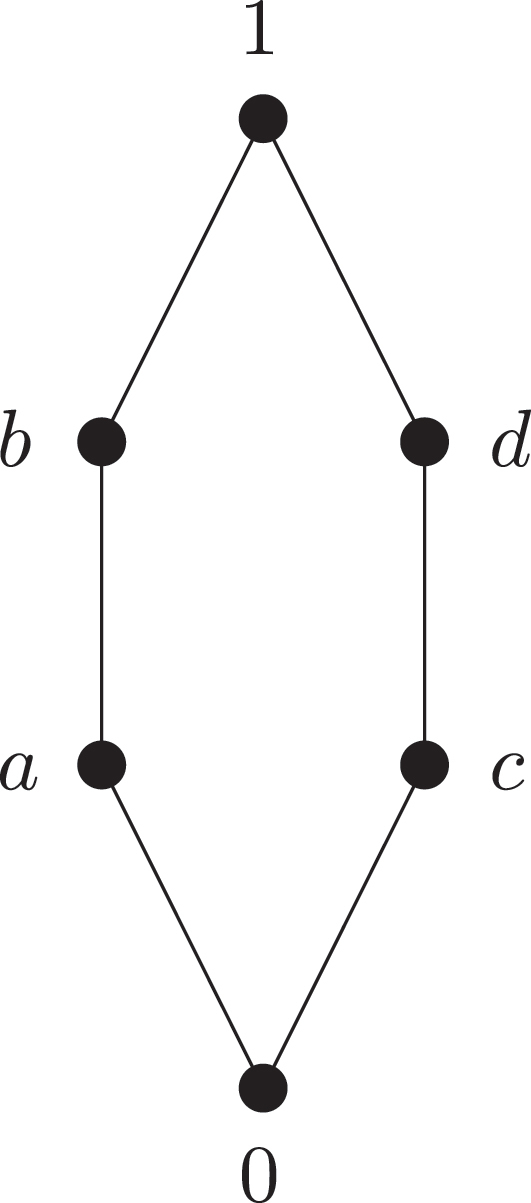
Ortholattice **O**_6_.

Theorem 2.3 shows that the non-modular ortholattice $O_{6}=\left(O_{6},\vee ,\wedge ,{\;}^{\prime }\right)$ depicted in Figure 4

satisfies identity (1.2) since **O**_6_ is the horizontal sum of two four-element chains where the antitone involution ′ is defined by

$$\begin{array}{ccccccc} x & 0 & a & b & c & d & 1\\ x^{\prime } & 1 & d & c & b & a & 0 \end{array}$$

Consider the lattice **O**_6_ once more, but define the complementation as follows:

$$\begin{array}{ccccccc} x & 0 & a & b & c & d & 1\\ x^{\prime } & 1 & c & d & a & b & 0 \end{array}$$

This operation is again an involution but it is not antitone. The resulting algebra will be denoted by $O_{6}^{*}$ . Again, $O_{6}^{*}$ satisfies identity (1.2).

**Example 4.**

**Figure 5:**
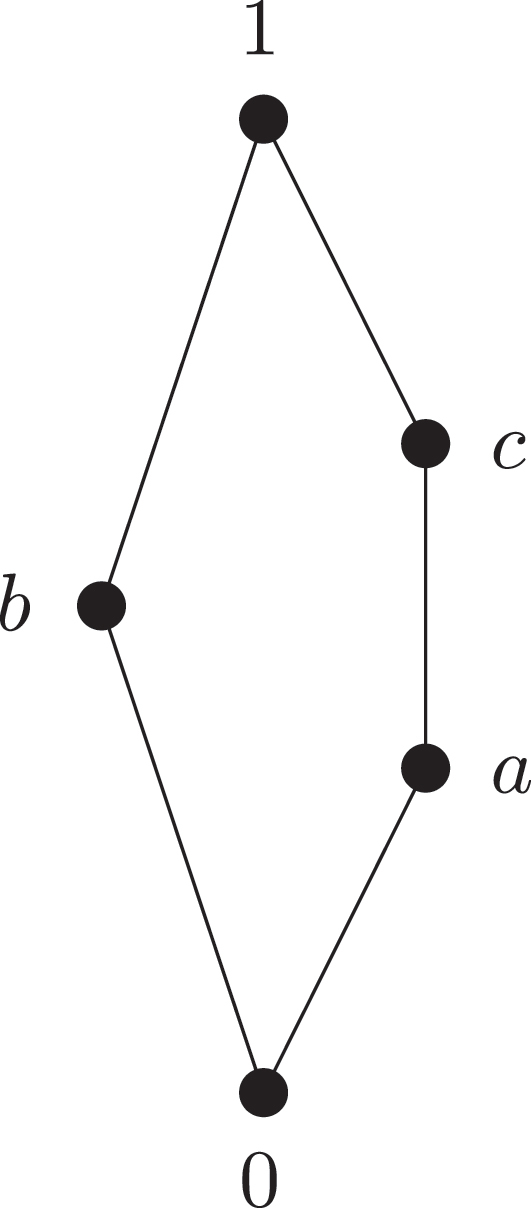
Non-modular lattice **N**_5_.

The non-modular lattice **N**_5_ = (*N*_5_, ∨, ∧) visualized in Figure 5.

is the horizontal sum of a three-element and a four-element chain. The complementation ′ defined by

$$\begin{array}{cccccc} x & 0 & a & b & c & 1\\ x^{\prime } & 1 & b & a & b & 1 \end{array}$$

is antitone, but not an involution and it does even not satisfy the identity *x* ≤ *x*″ since *c* ≰ *a* = *c*″. On the contrary, it satisfies the identity *x*″ ≤ *x*.

According to Theorem 2.3, $N_{5}=\left(N_{5},\vee ,\wedge ,{\;}^{\prime }\right)$ satisfies De Morgan’s laws and identity (1.2). One can easily check that **N**_5_ has just five congruences, namely Δ, *μ*, *α*, *β* and ∇ defined by

$$\begin{array}{ll} \Delta & ≔\left\{\left(x,x\right)|x\in N_{5}\right\},\\ \mu & ≔{\left\{0\right\}}^{2}\cup {\left\{a,c\right\}}^{2}\cup {\left\{b\right\}}^{2}\cup {\left\{1\right\}}^{2},\\ \alpha & ≔{\left\{0,b\right\}}^{2}\cup {\left\{a,c,1\right\}}^{2},\\ \beta & ≔{\left\{0,a,c\right\}}^{2}\cup {\left\{b,1\right\}}^{2},\\ \nabla & ≔N_{5}^{2}. \end{array}$$

**Figure 6:**
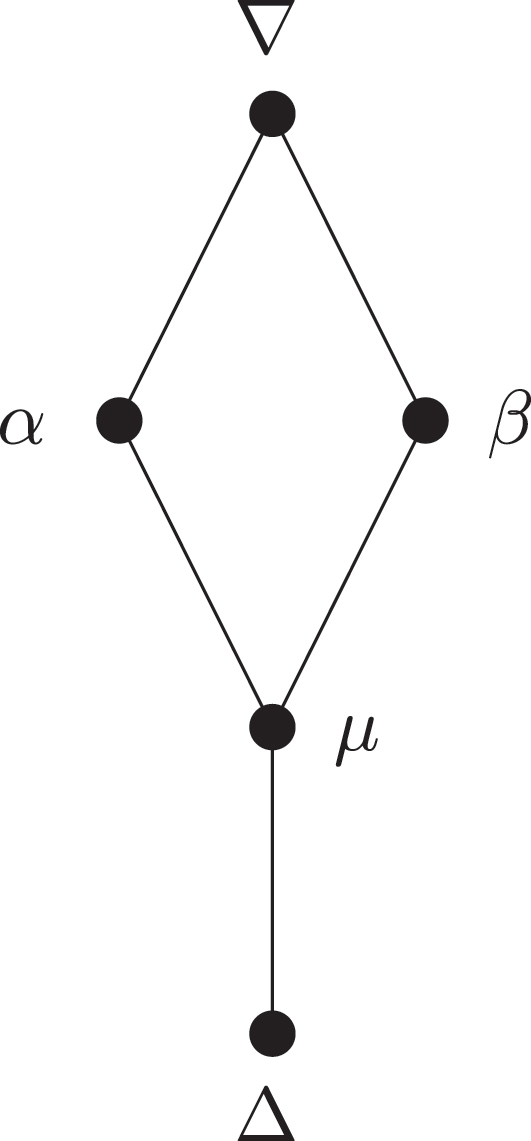
Congruence lattice of **N**_5_.

The congruence lattice of **N**_5_ is depicted in Figure 6.

Hence **N**_5_ is subdirectly irreducible. However, we can define the complementation in **N**_5_ also in a different way, namely as follows:

$$\begin{array}{cccccc} x & 0 & a & b & c & 1\\ x^{\prime } & 1 & b & c & b & 1 \end{array}$$

Then the resulting algebra $N_{5}^{*}$ satisfies the identities (1.2) and *x* ≤ *x*″ since *a* ≤ *c* = *a*″ = *c*″. The ortholattice **O**_6_ as well as the algebra $O_{6}^{*}$ has also five congruences, namely Δ, *μ*, *α*, *β* and ∇ defined by

$$\begin{array}{ll} \Delta & ≔\left\{\left(x,x\right)|x\in O_{6}\right\},\\ \mu & ≔{\left\{0\right\}}^{2}\cup {\left\{a,b\right\}}^{2}\cup {\left\{b^{\prime },a^{\prime }\right\}}^{2}\cup {\left\{1\right\}}^{2},\\ \alpha & ≔{\left\{0,a,b\right\}}^{2}\cup {\left\{b^{\prime },a^{\prime },1\right\}}^{2},\\ \beta & ≔{\left\{0,b^{\prime },a^{\prime }\right\}}^{2}\cup {\left\{a,b,1\right\}}^{2},\\ \nabla & ≔O_{6}^{2}. \end{array}$$

The congruence lattices of $N_{5}^{*}$ , **O**_6_ and $O_{6}^{*}$ coincide with that of **N**_5_ and hence also $N_{5}^{*}$ , **O**_6_ and $O_{6}^{*}$ are subdirectly irreducible.

The variety W of lattices with complementation satisfying identity (1.2) has several interesting subvarieties. Among these are the variety B of Boolean algebras and the varieties $V\left(N_{5}\right)$ , $V\left(N_{5}^{*}\right)$ , $V\left(O_{6}\right)$ and $V\left(O_{6}^{*}\right)$ generated by the corresponding algebras. These do not coincide because they satisfy different identities despite the fact that as lattices (without complementation) **N**_5_ and $N_{5}^{*}$ as well as **O**_6_ and $O_{6}^{*}$ are isomorphic.

It is worth noticing that also the ortholattice visualized in Figure 3 is subdirectly irreducible and hence it also generates a subvariety of the variety W which does not coincide with afore-mentioned ones.

By Jónsson Lemma, the two-element Boolean lattice **2** = {0, 1} is the only subdirectly irreducible member of B , **2** and **N**_5_ are the only subdirectly irreducible members of $V\left(N_{5}\right)$ , and **2** and **O**_6_ are the only subdirectly irreducible members of $V\left(O_{6}\right)$ .

Moreover, $V\left(O_{6}\right)$ satisfies the identity *x*″ ≈ *x* since the complementation in **2** as well as in **O**_6_ is an involution. On the other hand, $V\left(N_{5}\right)$ does not satisfy this identity, it satisfies only the identities *x*‴ ≈ *x*′ and *x*″ ≤ *x*.

That the varieties $V\left(N_{5}\right)$ and $V\left(O_{6}\right)$ both satisfy De Morgan’s laws follows from Theorem 2.3.

As pointed out already by Birkhoff [9], if **A** is a subdirectly irreducible algebra and V(A) denotes the variety generated by **A** then the cardinality of the free algebra $F_{V\left(A\right)}\left(n\right)$ in V(A) with *n* free generators can be estimated as follows:

(2.1) $$|F_{V\left(A\right)}\left(n\right)|\leq |A{|}^{|A{|}^{n}}.$$

However, Berman [10] used the estimation $|F_{V\left(A\right)}\left(n\right)|\leq |A{|}^{k}$ and got a method how to determine a “good” number *k*. The process may not give a minimal *k* in general, though in congruence-distributive varieties it should. E. W. Kiss and R. Freese developed a computer program accessible at the address

http://www.cs.elte.hu/^∼^ewkiss/software/naprog

computing the optimal estimation of *k*. Of course, the varieties $V\left(N_{5}\right)$ and $V\left(O_{6}\right)$ are locally finite and the cardinalities of the corresponding free algebras and estimations for *k* computed by this program are as follows:

For $V\left(N_{5}\right)$ we have

$$\begin{array}{ccc} n & \left|F_{V\left(N_{5}\right)}\left(n\right)\right| & k\\ 1 & 5 & 1\\ 2 & 152 & 9\\ 3 & \geq 100,036 & 61\\ 4 & & 369\\ 5 & & 2,101 \end{array}$$

and for $V\left(O_{6}\right)$

$$\begin{array}{ccc} n & \left|F_{V\left(O_{6}\right)}\left(n\right)\right| & k\\ 1 & 4 & 2\\ 2 & 100 & 4\\ 3 & \geq 249,275 & 48\\ 4 & & 400\\ 5 & & 2,880 \end{array}$$

The same tables hold for $N_{5}^{*}$ and $O_{6}^{*}$ , respectively.

## 3 Identities of symmetric differences

Associativity of operations in orthomodular lattices was treated also by Matoušek [3] and Navara and his collaborators [1], [2].

As mentioned in the introduction, it is well-known (see e.g. [10]) that in a Boolean lattice (*L*, ∨, ∧, ′, 0, 1) the term operations +_1_ and +_2_ coincide and these operations are associative, i.e. they satisfy the identities

$$\left(x{+}_{1}y\right){+}_{1}z\approx x{+}_{1}\left(y{+}_{1}z\right)\text{ and }\left(x{+}_{2}y\right){+}_{2}z\approx x{+}_{2}\left(y{+}_{2}z\right),$$

respectively. The question arises whether, conversely, in a lattice with complementation, associativity of +_1_ or +_2_ implies lattice distributivity. We will show that this is indeed the case.

At first we prove an auxiliary result using identities similar to those studied in refs. [4], [5].

**Lemma 3.1.**

Let (*L*, ∨, ∧, ′, 0, 1) be a lattice with complementation. Then the following are equivalent:

(i) *x* ∧ *y* ≈ *x* ∧ (*x*′ ∨ *y*),
(ii) *x* ∨ *y* ≈ *x* ∨ (*x*′ ∧ *y*).

**Proof.**

Assume (i). Then, by Lemma 1.1 we have *x*″ ≈ *x*, and from (i) we get

(3.1) $$x^{\prime }\wedge y\approx x^{\prime }\wedge \left(x^{\prime \prime }\vee y\right)\approx x^{\prime }\wedge \left(x\vee y\right).$$

Using this we obtain

$${\left(x\vee y\right)}^{\prime }\wedge x^{\prime }\approx {\left(x\vee y\right)}^{\prime }\wedge \left(\left(x\vee y\right)\vee x^{\prime }\right)\approx {\left(x\vee y\right)}^{\prime }\wedge 1\approx {\left(x\vee y\right)}^{\prime }.$$

Thus

(3.2) $${\left(x\vee y\right)}^{\prime }\wedge x^{\prime }\approx {\left(x\vee y\right)}^{\prime }$$

and hence

$$x\vee {\left(x^{\prime }\vee y\right)}^{\prime }\approx x\vee \left({\left(x^{\prime }\vee y\right)}^{\prime }\wedge x^{\prime \prime }\right)\approx x\vee \left({\left(x^{\prime }\vee y\right)}^{\prime }\wedge x\right)\approx x$$

showing

(3.3) $$x\approx x\vee {\left(x^{\prime }\vee y\right)}^{\prime }.$$

Using (i) we get

$$x\wedge \left(y\vee {\left(y\vee x^{\prime }\right)}^{\prime }\right)\approx x\wedge \left(x^{\prime }\vee y\vee {\left(y\vee x^{\prime }\right)}^{\prime }\right)\approx x\wedge 1\approx x$$

and hence

$$x\approx x\wedge \left(y\vee {\left(y\vee x^{\prime }\right)}^{\prime }\right).$$

Using this and (3.3) we obtain

$$x\vee {\left(x\vee y^{\prime }\right)}^{\prime }\approx \left(x\vee {\left(x\vee y^{\prime }\right)}^{\prime }\right)\vee \left(\left(x\vee {\left(x\vee y^{\prime }\right)}^{\prime }\right)\wedge y\right)\approx \left(x\vee {\left(x\vee y^{\prime }\right)}^{\prime }\right)\vee y\approx x\vee \left({\left(x\vee y^{\prime }\right)}^{\prime }\vee y\right)\approx x\vee y.$$

Thus

(3.4) $$x\vee y\approx x\vee {\left(x\vee y^{\prime }\right)}^{\prime }.$$

Using this and (3.1) we get

$$\left(x\vee y\right)\wedge x^{\prime }\approx \left(x\vee {\left(x\vee y^{\prime }\right)}^{\prime }\right)\wedge x^{\prime }\approx {\left(x\vee y^{\prime }\right)}^{\prime }\wedge x^{\prime }.$$

Thus

$$\left(x\vee y\right)\wedge x^{\prime }\approx {\left(x\vee y^{\prime }\right)}^{\prime }\wedge x^{\prime }.$$

From this, (3.2) and (3.1) we obtain

$${\left(x\vee y\right)}^{\prime }\approx {\left(x\vee y\right)}^{\prime }\wedge x^{\prime }\approx {\left(x\vee y^{\prime \prime }\right)}^{\prime }\wedge x^{\prime }\approx \left(x\vee y^{\prime }\right)\wedge x^{\prime }\approx x^{\prime }\wedge y^{\prime }.$$

Thus

$${\left(x\vee y\right)}^{\prime }\approx x^{\prime }\wedge y^{\prime }.$$

Finally, using this and (3.4) we get

$$x\vee y\approx x\vee {\left(x\vee y^{\prime }\right)}^{\prime }\approx x\vee \left(x^{\prime }\wedge y^{\prime \prime }\right)\approx x\vee \left(x^{\prime }\wedge y\right).$$

Therefore (ii) holds. On the other hand, if (ii) holds, then again *x*″ ≈ *x* by Lemma 1.1. The proof that (ii) implies (i) follows by dualizing the preceding arguments. □

Now we get a connection of the afore-mentioned identities with distributivity of the lattice in question. This is an essential result for the proof of our next theorem.

**Theorem 3.1.**

Let **L** = (*L*, ∨, ∧, ′, 0, 1) be a lattice with complementation satisfying one of the following identities

(i) *x* ∧ *y* ≈ *x* ∧ (*x*′ ∨ *y*),
(ii) *x* ∨ *y* ≈ *x* ∨ (*x*′ ∧ *y*).

Then **L** is distributive and hence Boolean.

**Proof.**

According to Lemmas 1.1 and 3.1 we have *x*″ ≈ *x*, and (i) and (ii) are equivalent. Therefore we may assume that both identities are satisfied. Using these identities we get

$$x\wedge \left(y\vee z\right)\approx x\wedge \left(x^{\prime }\vee y\vee z\right)\approx x\wedge \left(x^{\prime }\vee \left(x\wedge y\right)\vee z\right)\approx x\wedge \left(\left(x\wedge y\right)\vee z\right).$$

Thus

(3.5) $$x\wedge \left(y\vee z\right)\approx x\wedge \left(\left(x\wedge y\right)\vee z\right).$$

Using this we obtain

$$\begin{array}{ll} x\wedge \left(y\vee z\right) & \approx \left(x\wedge \left(y\vee z\right)\right)\vee \left(x\wedge \left(y\vee z\right)\wedge y\right)\\ & \approx \left(x\wedge \left(y\vee z\right)\right)\vee \left(x\wedge \left(\left(x\wedge y\right)\vee z\right)\wedge y\right)\\ & \approx \left(x\wedge \left(y\vee z\right)\right)\vee \left(\left(x\wedge y\right)\wedge \left(\left(x\wedge y\right)\vee z\right)\right)\approx \left(x\wedge \left(y\vee z\right)\right)\vee \left(x\wedge y\right) \end{array}$$

and hence

$$x\wedge \left(y\vee z\right)\approx \left(x\wedge \left(y\vee z\right)\right)\vee \left(x\wedge y\right).$$

Finally, using this, (ii), (3.5) and (i) we get

$$\begin{array}{ll} x\wedge \left(y\vee z\right) & \approx \left(x\wedge \left(y\vee z\right)\right)\vee \left(x\wedge y\right)\approx \left(x\wedge y\right)\vee \left({\left(x\wedge y\right)}^{\prime }\wedge \left(x\wedge \left(y\vee z\right)\right)\right)\\ & \approx \left(x\wedge y\right)\vee \left({\left(x\wedge y\right)}^{\prime }\wedge x\wedge \left(\left(x\wedge y\right)\vee z\right)\right)\\ & \approx \left(x\wedge y\right)\vee \left({\left(x\wedge y\right)}^{\prime }\wedge \left(\left(x\wedge y\right)\vee z\right)\wedge x\right)\approx \left(x\wedge y\right)\vee \left({\left(x\wedge y\right)}^{\prime }\wedge z\wedge x\right)\\ & \approx \left(x\wedge y\right)\vee \left(x\wedge z\right). \end{array}$$

□

Our main result concerning the associativity of the symmetric difference is as follows.

**Theorem 3.2.**

Let **L** = (*L*, ∨, ∧, ′, 0, 1) be a lattice with complementation. Then **L** is Boolean if and only if +_1_ or +_2_ is associative.

**Proof.**

If **L** is Boolean then +_1_ = +_2_ and this operation is associative (see [7]). Conversely, assume either +_1_ or +_2_ to be associative. Let + ∈ {+_1_, +_2_}. Then

$$x+0\approx x,x+1\approx x^{\prime }\text{ and }x+x\approx 0$$

and hence

$${\left(x+y\right)}^{\prime }\approx x+\left(y+1\right)\approx x+y^{\prime }\approx x^{\prime }+y\text{ and }x^{\prime \prime }\approx x+1+1\approx x+0\approx x.$$

Thus

$${\left(x+y\right)}^{\prime }\approx x+y^{\prime }\approx x^{\prime }+y\text{ and }x^{\prime \prime }\approx x.$$

For +_1_ we have

(3.6) $$x\approx \left(x{+}_{1}y\right){+}_{1}y\approx \left(y\wedge {\left(x{+}_{1}y\right)}^{\prime }\right)\vee \left(y^{\prime }\wedge \left(x{+}_{1}y\right)\right).$$

Using this we obtain

$$\begin{array}{ll} x\wedge {\left(x{+}_{1}y\right)}^{\prime } & \approx x\wedge {\left(x{+}_{1}y\right)}^{\prime }\wedge \left(\left(x\wedge {\left(x{+}_{1}y\right)}^{\prime }\right)\vee \left(x^{\prime }\wedge \left(x{+}_{1}y\right)\right)\right)\approx x\wedge {\left(x{+}_{1}y\right)}^{\prime }\wedge y\\ & \approx \left(x\wedge y\right)\wedge \left(x^{\prime }{+}_{1}y\right)\approx \left(x\wedge y\right)\wedge \left(\left(x\wedge y\right)\vee \left(x^{\prime }\wedge y^{\prime }\right)\right)\approx x\wedge y. \end{array}$$

Thus

$$x\wedge {\left(x{+}_{1}y\right)}^{\prime }\approx x\wedge y.$$

From this and (3.6) we get

$$\begin{array}{ll} x & \approx \left(y\wedge {\left(x{+}_{1}y\right)}^{\prime }\right)\vee \left(y^{\prime }\wedge \left(x{+}_{1}y\right)\right)\approx \left(x\wedge y\right)\vee \left(y^{\prime }\wedge \left(x{+}_{1}y^{\prime \prime }\right)\right)\\ & \approx \left(x\wedge y\right)\vee \left(y^{\prime }\wedge {\left(x{+}_{1}y^{\prime }\right)}^{\prime }\right)\approx \left(x\wedge y\right)\vee \left(x\wedge y^{\prime }\right). \end{array}$$

Thus

$$x\approx \left(x\wedge y\right)\vee \left(x\wedge y^{\prime }\right).$$

Using this we obtain

$$x^{\prime }\vee y\approx \left(x^{\prime }\wedge y\right)\vee \left(x^{\prime }\wedge y^{\prime }\right)\vee \left(x\wedge y\right)\vee \left(x^{\prime }\wedge y\right)\approx \left(x^{\prime }\wedge y\right)\vee \left(x^{\prime }\wedge y^{\prime }\right)\vee \left(x\wedge y\right)\approx x^{\prime }\vee \left(x\wedge y\right).$$

Thus *x*′ ∨ *y* ≈ *x*′ ∨ (*x* ∧ *y*) and, consequently, *x* ∨ *y* ≈ *x* ∨ (*x*′ ∧ *y*). By Theorem 3.1 this implies that **L** is Boolean. For +_2_ we can use similar arguments. We have

(3.7) $$x\approx \left(x{+}_{2}y\right){+}_{2}y\approx \left(y\vee \left(x{+}_{2}y\right)\right)\wedge \left(y^{\prime }\vee {\left(x{+}_{2}y\right)}^{\prime }\right).$$

Using this we get

$$\begin{array}{ll} x\vee \left(x{+}_{2}y\right) & \approx \left(x\vee \left(x{+}_{2}y\right)\right)\vee \left(\left(x\vee \left(x{+}_{2}y\right)\right)\wedge \left(x^{\prime }\vee {\left(x{+}_{2}y\right)}^{\prime }\right)\right)\approx x\vee \left(x{+}_{2}y\right)\vee y\\ & \approx \left(x\vee y\right)\wedge \left(x{+}_{2}y\right)\approx \left(x\vee y\right)\vee \left(\left(x\vee y\right)\wedge \left(x^{\prime }\vee y^{\prime }\right)\right)\approx x\vee y. \end{array}$$

Thus

$$x\vee \left(x{+}_{2}y\right)\approx x\vee y.$$

From this and (3.7) we obtain

$$x\approx \left(y\vee \left(x{+}_{2}y\right)\right)\wedge \left(y^{\prime }\vee {\left(x{+}_{2}y\right)}^{\prime }\right)\approx \left(x\vee y\right)\wedge \left(y^{\prime }\vee \left(x{+}_{2}y^{\prime }\right)\right)\approx \left(x\vee y\right)\wedge \left(x\vee y^{\prime }\right).$$

Thus

$$x\approx \left(x\vee y\right)\wedge \left(x\vee y^{\prime }\right)$$

and hence

$$x\wedge y\approx \left(x\vee y\right)\wedge \left(x\vee y^{\prime }\right)\wedge \left(x\vee y\right)\wedge \left(x^{\prime }\vee y\right)\approx \left(x\vee y\right)\wedge \left(x\vee y^{\prime }\right)\wedge \left(x^{\prime }\vee y\right)\approx x\wedge \left(x^{\prime }\vee y\right).$$

This shows *x* ∧ *y* ≈ *x* ∧ (*x*′ ∨ *y*) which, by Theorem 3.1, implies that **L** is Boolean. □

It is worth noticing that if the lattice **L** in Theorem 3.2 is assumed to be orthomodular, then an alternative proof of the assertion in Theorem 3.2 can be found in ref. [3] the reference to Proposition 4.3 is meant to be [12, Proposition 4.3], and similarly, Proposition 4.2.1 is meant to be [13, Proposition 4.2.1]. [11], [12], [13].

As shown above, associativity of the symmetric difference in a lattice **L** with complementation yields distributivity of **L**. Surprisingly, also a simple identity in only two variables formulated for the symmetric difference yields the same, see the following result.

**Theorem 3.3.**

Let **L** = (*L*, ∨, ∧, ′, 0, 1) be a lattice with complementation. Then **L** is a Boolean algebra if and only if + satisfies the identity

(x+y)+y≈x

where either + = +_1_ or + = +_2_.

**Proof.**

Of course, if **L** is a Boolean algebra then according to Theorem 3.2 the symmetric difference is associative and hence it satisfies (*x* + *y*) + *y* ≈ *x* + (*y* + *y*) ≈ *x* + 0 ≈ *x*. Conversely, assume **L** to satisfy the identity (*x* + *y*) + *y* ≈ *x*. If **L** satisfies the identity (*x* +_1_ *y*) +_1_ *y* ≈ *x* then

$$\begin{array}{ll} x^{\prime }\wedge y & \approx \left(x^{\prime }\wedge y\right)\wedge \left(\left(x^{\prime }\wedge y\right)\vee \left(x\wedge y^{\prime }\right)\right)\approx x^{\prime }\wedge y\wedge \left(x{+}_{1}y\right)\\ & \approx x^{\prime }\wedge \left(\left(y{+}_{1}x\right){+}_{1}x\right)\wedge \left(x{+}_{1}y\right)\\ & \approx \left(x^{\prime }\wedge \left(x{+}_{1}y\right)\right)\wedge \left(\left({\left(y{+}_{1}x\right)}^{\prime }\wedge x\right)\vee \left(\left(y{+}_{1}x\right)\wedge x^{\prime }\right)\right)\approx x^{\prime }\wedge \left(x{+}_{1}y\right) \end{array}$$

and hence

$$x\vee y\approx x\vee \left(\left(y{+}_{1}x\right){+}_{1}x\right)\approx x\vee \left({\left(y{+}_{1}x\right)}^{\prime }\wedge x\right)\vee \left(\left(y{+}_{1}x\right)\wedge x^{\prime }\right)\approx x\vee \left(x^{\prime }\wedge \left(x{+}_{1}y\right)\right)\approx x\vee \left(x^{\prime }\wedge y\right)$$

which implies that **L** is Boolean according to Theorem 3.1. If **L** satisfies the identity (*x* +_2_ *y*) +_2_ *y* ≈ *x* then

$$\begin{array}{lc} x\vee y & \approx \left(x\vee y\right)\vee \left(\left(x\vee y\right)\wedge \left(x^{\prime }\vee y^{\prime }\right)\right)\approx \left(x\vee y\right)\vee \left(x{+}_{2}y\right)\\ & \approx x\vee \left(\left(y{+}_{2}x\right){+}_{2}x\right)\vee \left(x{+}_{2}y\right)\\ & \approx x\vee \left(x{+}_{2}y\right)\vee \left(\left(\left(y{+}_{2}x\right)\vee x\right)\wedge \left({\left(y{+}_{2}x\right)}^{\prime }\vee x^{\prime }\right)\right)\approx x\vee \left(x{+}_{2}y\right) \end{array}$$

and hence

$$\begin{array}{ll} x^{\prime }\wedge y & \approx x^{\prime }\wedge \left(\left(y{+}_{2}x\right){+}_{2}x\right)\approx x^{\prime }\wedge \left(\left(y{+}_{2}x\right)\vee x\right)\wedge \left({\left(y{+}_{2}x\right)}^{\prime }\vee x^{\prime }\right)\\ & \approx x^{\prime }\wedge \left(x\vee \left(x{+}_{2}y\right)\right)\approx x^{\prime }\wedge \left(x\vee y\right) \end{array}$$

which because of

$$\begin{array}{ll} x^{\prime } & \approx 1\wedge \left(x^{\prime }\vee 0\right)\approx \left(x\vee 1\right)\wedge \left(x^{\prime }\vee 1^{\prime }\right)\approx x{+}_{2}1,\\ x^{\prime \prime } & \approx \left(x{+}_{2}1\right){+}_{2}1\approx x \end{array}$$

implies *x* ∧ *y* ≈ *x*″ ∧ *y* ≈ *x*″ ∧ (*x*′ ∨ *y*) ≈ *x* ∨ (*x*′ ∨ *y*) proving **L** to be Boolean according to Theorem 3.1. □

In ref. [5] it was shown that a lattice (*L*, ∨, ∧, ′) with a unary operation ′ is Boolean if and only if it satisfies the identities

$$x^{\prime }\vee \left(x\wedge y\right)\approx x^{\prime }\vee y\text{ and }x^{\prime }\wedge \left(x\vee y\right)\approx x^{\prime }\wedge y.$$

In ref. [4] single identities are presented that force a lattice with a unary operation to be Boolean. Finally, in ref. [14] it was proved that a lattice (*L*, ∨, ∧, ′) with a unary operation ′ is Boolean if and only if it satisfies the identity

$$\left(x\wedge y\right)\vee \left(x\wedge y^{\prime }\right)\approx \left(x\vee y\right)\wedge \left(x\vee y^{\prime }\right)$$

(Proposition 4.2.1).

## 4 De Morgan’s laws

It is well-known that a lattice (*L*, ∨, ∧, ′) with an antitone involution ′ satisfies De Morgan’s laws

$${\left(x\vee y\right)}^{\prime }\approx x^{\prime }\wedge y^{\prime }\text{ and }{\left(x\wedge y\right)}^{\prime }\approx x^{\prime }\vee y^{\prime }.$$

However, De Morgan’s laws may be satisfied also in the case that ′ is not an involution.

Now we are interested in conditions under which a lattice with a unary operation ′ satisfies De Morgan’s laws. At first, we see that the lattice **N**_5_ with the complementation defined in Example 4 satisfies De Morgan’s laws despite the fact that the complementation ′ is not an involution.

**Example 5.**

The complementation ′ of the non-modular lattices **N**_5_ and $N_{5}^{*}$ is antitone, but not an involution, but these lattices still satisfy De Morgan’s laws. The complementation ′ of the non-modular lattice **O_6_** is antitone and an involution and satisfies De Morgan’s laws. The complementation ′ of the non-modular lattice $O_{6}^{*}$ is not antitone, but it is an involution, and does not satisfy De Morgan’s laws.

**Theorem 4.1.**

Let **L** = (*L*, ∨, ∧, ′) be a lattice with a unary operation ′. Then the following are equivalent:

(i) **L** satisfies the identities (*x* ∨ *y*)′ ≈ *x*′ ∧ *y*′ and (*x* ∧ *y*)′ ≈ *x*′ ∨ *y*′,
(ii) the operation ′ is antitone and **L** satisfies the identities (*x* ∨ *y*)′ ∧ *x*′ ≈ *x*′ ∧ *y*′ and (*x* ∧ *y*)′ ∨ *x*′ ≈ *x*′ ∨ *y*′.

**Proof.**

We prove that the following are equivalent:

(iii) **L** satisfies the identity (*x* ∨ *y*)′ ≈ *x*′ ∧ *y*′,
(iv) the operation ′ is antitone and **L** satisfies the identity (*x* ∨ *y*)′ ∧ *x*′ ≈ *x*′ ∧ *y*′.

The rest follows by duality. Let *a*, *b* ∈ *L*.

(iii) ⇒ (iv):

If *a* ≤ *b* then *b*′ = (*a* ∨ *b*)′ = *a*′ ∧ *b*′ ≤ *a*′ showing that ′ is antitone. Moreover, (*a* ∨ *b*)′ ∧ *a*′ = *a*′ ∧ *b*′ ∧ *a*′ = *a*′ ∧ *b*′ proving (iv).

(iv) ⇒ (iii):

Because of *a* ≤ *a* ∨ *b* and *b* ≤ *a* ∨ *b* we have (*a* ∨ *b*)′ ≤ *a*′ and (*a* ∨ *b*)′ ≤ *b*′ and hence (*a* ∨ *b*)′ ≤ *a*′ ∧ *b*′. Conversely, (iv) implies *a*′ ∧ *b*′ = (*a* ∨ *b*)′ ∧ *a*′ ≤ (*a* ∨ *b*)′ which together with (*a* ∨ *b*)′ ≤ *a*′ ∧ *b*′ yields (*a* ∨ *b*)′ = *a*′ ∧ *b*′. □

**Example 6.**

One can easily check that condition (ii) of Theorem 4.1 is satisfied in the lattices **N**_5_, $N_{5}^{*}$ and **O**_6_, but not in the lattice $O_{6}^{*}$ .
